# Antidiarrheal Activity of *Dialium guineense* Willd Fruit Pulp in Wistar Rats

**DOI:** 10.1155/2022/4161714

**Published:** 2022-10-22

**Authors:** Tcharé Assiki, Aboudoulatif Diallo, Essotolom Badjabaïssi, Mindédé Assih, Tchazou Kpatcha, Kokou Idoh, Kossivi Dosseh, Amegnona Agbonon

**Affiliations:** ^1^Laboratory of Toxicology, Faculty of Health Sciences, University of Lomé (Togo), Togo; ^2^Laboratory of Pharmacology, Faculty of Health Sciences, University of Lomé (Togo), Togo; ^3^Department of Animal Physiology, Faculty of Sciences, University of Lomé (Togo), Togo

## Abstract

**Objective:**

This study is aimed at evaluating the effects of *Dialium guineense* Willd fruit pulp powder on diarrhea induced by castor oil in Wistar rats.

**Materials and Methods:**

Three different tests were carried out. A preventive test by administration of a single dose of 250, 500, 1000, 2000, and 4000 mg/kg before the induction of diarrhea by castor oil. Another preventive test after repeated administration of *Dialium guineense* at 250, 500, and 1000 mg/kg/day for 8 days, before the induction of diarrhea, was done. The third test was a curative test with a single dose of 250, 500, 1000, and 2000 mg/kg after the induction of diarrhea by castor oil.

**Results:**

*D. guineense* fruit pulp at 1000, 2000, and 4000 mg/kg administered before the induction of diarrhea, has significantly delayed diarrhea; reduced the frequency of defecation, reduced the amount of diarrheal stools, and also reduced the purging index, with a degree of inhibition comparable to that of loperamide. But the water content of the stools of the group treated with *D. guineense* does not change significantly compared to the controls. *D. guineense* has reduced significantly from 500 mg/kg the diarrhea induced by castor oil after 8 days of treatment. It appears that the doses of 250 and 500 mg/kg, which were not effective with the single-dose preventive test, significantly delayed diarrhea; reduces the frequency of diarrheal stools and also reduces the purging index. *D. guineense* administered, after the induction of diarrhea, by castor oil has significantly reduced the diarrhea from 250 mg/kg.

**Conclusion:**

The fruit pulp of *D. guineense* has showed antidiarrheal activities in Wistar rats by reducing the frequency of defecation, the amount of diarrheal fecal matter emitted as well as the water content. It also delayed the onset of diarrhea and significantly reduced the purging index like loperamide.

## 1. Introduction

Developing countries are especially prone to the risk of these diseases, including diarrhea, influenza, and microbial infections [1]. Among these, diarrhea represents a sizeable burden, being regarded as the second most common cause of death in the world [2]. It has been reported that between 2 and 4 billion cases of diarrhea occur in poor countries each year, with children (age<5) particularly susceptible. The World Health Organization (WHO) has recommended several approaches to alleviate the burden of diarrheal illness, of which a cornerstone is oral rehydration therapy. Of the established conventional antidiarrheal drugs, many are associated with adverse effects and contraindications [3]. For their treatment, humans are largely dependent on plants [4, 5], especially in Africa [6] where more than eighty percent of the population relies exclusively on plants for healing [7].

*Dialium guineense* is a shrub of the family Leguminosae. It has a straight, grayish, and smooth stem. The pulp of the fruits of *D. guineense* is appreciated by all socioeconomic strata and all Togolese age groups. It contains huge amounts of nutrients which are very beneficial for the body [8]. Its stem bark is used for the treatment of cough, toothache, and bronchitis [9]. Many studies have showed the antiulcer activity (leaves) [10], antimicrobial activity (fruits, leaves, roots, stem bark) [11, 12], antivibrio activity (leaves) [13], antiplasmodium activity (leaves) [14, 15], molluscicidal activity (fruit) [16], analgesic activity (bark) [17], antihepatotoxic activity (fruit pulp) [18], antidiarrheal activity (leaves) [19], and antioxidant activity (fruits; leaves, stem bark) [20, 21].

Because of the sweetness of this fruit, *D. guineense* is widely consumed by the Togolese population. A significant portion of consumers complains of constipation after taking this pulp. Therefore, this study is aimed at evaluating the antidiarrheal activities of the fruit pulp of *D. guineense*. More specifically, to evaluate the preventive effects of this pulp in single and repeated doses on diarrhea induced by castor oil as well as its curative effects on the same diarrhea in Wistar rats.

## 2. Materials and Methods

### 2.1. Animals

Wistar rats of 200-250 g (4 to 5 months old) were used. They were obtained for the animal facility center of the Department of Physiology and Pharmacology of the Faculty of Sciences of the University of Lomé (Togo). These animals were treated according to the standards of the Organization for Economic Cooperation and Development (OECD) [22]. They were kept at a temperature of 23 ± 3°C and on a 12-h light/dark cycle with free access to water and food (except on days experience) in pharmaceuticals sciences department animal facility. Animals were acclimatized to laboratory conditions for one week before each experiment. All studies were done in the Toxicology Laboratory at the Faculty of Health Sciences, University of Lomé (Togo).

### 2.2. Plant Material and Chemicals

The plant material is the fruit pulp of *D. guineense*. These fruits were bought at the “Agoè Assiyéyé” market, a local market, from ladies from Mission Tové, a village located about twenty kilometers north of the city of Lomé. A voucher specimen was kept in the herbarium of the Laboratory of Botany and Plant Ecology (Faculty of Science/University of Lomé) under the reference number Togo 15879. Fruits dark-colored hard coats are broken to expose the soft pink pulp. The pulp was peeled and then dried at room temperature by hot air oven (Amstel Hearson Oven, England) to evaporate its water content to a thick orange paste. Dried pulp was blended to powder. Loperamide (Denk-Pharma), a synthetic opioid agonist was used as a positive control in the inhibition of diarrhea. Castor oil (Cooper) was used in this study for the induction of diarrhea. This oil releases ricinoleic acid in the digestive tract which causes diarrhea through mechanisms involving inflammation of the gastrointestinal mucosa [23, 24].

### 2.3. Preparation of *D. Guineense* Fruit Powder

*D. guineense* fruit powder was treated according to the method used by Odukoya et al. [16]. These fruits were manually cleaned to remove waste as well as unripe fruits. The healthy fruits obtained are shelled by hand to free them from their pods. They are then always scraped manually to separate the pulp from the seeds. The pulp thus obtained is dried under air conditioning and then reduced to powder. This powder is stored at -20°C until use.

### 2.4. Antidiarrheal Tests

#### 2.4.1. Evaluation of the Preventive Effects of Single-Dose of *D. Guineense* Fruit Pulp on Castor Oil Induced Diarrhea

The method of Abdelaand and Njume and Godukawith some modifications were used for this study [23, 25]. Thirty-five rats of 200-250 g of both sexes were fasted for 18 h before the test. They had free access to water. They were divided into seven groups of five animals. The first group (group 1) received distilled water (negative contSrol), the second group (group 2) received 3 mg/kg of loperamide (positive control), and the 3^rd^, 4^th^, 5^th^, 6^th^, and 7^th^ groups received, respectively, 250, 500, 1000, 2000, and 4000 mg/kg. *D. guineense* fruit powder pulp dissolved in distilled water. The high doses of 1000, 2000, and 4000 mg/kg are used in order to check if at very high doses *D. guineense* fruit powder pulp would not cause a paralytic ileus.

One hundred and twenty minutes after the drug treatment, each animal received orally castor oil at 10 ml/kg of body weight and was immediately placed in a metabolic cage containing white absorbent paper whose mass is known in advance. The latency period (time separating the induction of diarrhea and the appearance of the first diarrheal drop) was recorded. Observation of defecation was continued for 4 h on the paper, which was replaced every 1 h after recording the number of defecations. Paper weight was also recorded. The paper is then dried under air conditioning until it is completely dehydrated. The dry mass was also recorded. The percentage of rats that responded to diarrhea (Equation (1)), stool water content (Equation (2)), percentage inhibition of diarrhea (Equation (3)), and purging index (Equation (4)), were determined by the following formulas:

(1) $$\begin{array}{c} \text{Percentage of rats that responded to diarrhea}=\frac{\text{number of respondants }}{\text{number of the rat in the group}}\text{x}100, \end{array}$$

(2) $$\begin{array}{c} \text{Stool water content}=\frac{\text{fresh mass}-\text{dry mass}}{\text{fresh mass}}\text{x}100, \end{array}$$

(3) $$\begin{array}{c} \text{Percentageinhibition of diarrhea}=\frac{\text{Mean number of faeces of NC}-\text{Mean number of the TG }}{\text{Mean number of faeces of NC}}\text{x}100, \end{array}$$

(NC: negative control; TG: test group)

(4) $$\begin{array}{c} \text{Purging index}=\frac{\%\text{of respondants }x\text{ mean number of stool}}{\text{Mean latent period}}. \end{array}$$

#### 2.4.2. Evaluation of the Preventive Effects of Repeated Administration of *D. Guineense* Fruit Pulp on Castor Oil Induced Diarrhea

The method of Vanjari et al. was used with some modifications [26]. Twenty-five rats weighing 200 to 250 g were divided into five groups of five rats. Each group received daily, for 7 days, the following treatment. The first group (group 1) received distilled water during the seven days (negative control). The second group (group 2) received 1 mg/kg of loperamide dissolved in distilled water (positive control). The 3^rd^, 4^th^, and 5^th^ groups received, respectively, 250, 500, and 1000 mg/kg of *D. guineense* fruit pulp dissolved in distilled water.

On the eighth day, all animals were fasted for 12 h with free access to water until one hour before the experiment. They then received the following treatment: the first group (group 1) received distilled water (negative control), the second group (group 2) received 1 mg/kg of loperamide dissolved in water distilled (positive control). The 3^rd^, 4^th^, and 5^th^ groups received, respectively, 250, 500, and 1000 mg/kg of *D. guineense* fruit powder pulp dissolved in distilled water. One hundred and twenty minutes after this treatment, all the rats received 10 ml/kg of body weight of castor oil orally. They were immediately distributed in metabolic cages containing white absorbent paper. Previous diarrheal parameters were also assessed.

#### 2.4.3. Evaluation of the Curative Effects of *D. Guineense* Fruit Pulp on Castor Oil-Induced Diarrhea

The method of Tagne et al. was used with some modifications [27]. Thirty Wistar rats weighing 250 to 300 g of both sexes were fasted for 18 h with free access to water and were divided into six groups of five rats. Castor oil was subsequently administered to all rats at a rate of 10 ml/kg (for the induction of diarrhea).

Twenty minutes after diarrhea induction, all groups received specific treatment as follows. The first group (group 1) received distilled water (negative control). The second group (group 2) received 3 mg/kg of loperamide dissolved in distilled water (positive control). The 3^rd^, 4^th^, 5^th^, and 6^th^group, respectively, received orally 250, 500, 1000, and 2000 mg/kg of *D. guineense* fruit pulp dissolved in distilled water. They were immediately placed in metabolic cages containing white absorbent paper. The observation of the rats was made for 4 h. The paper placed in the metabolic cage being renewed every hour, and the diarrheal parameters were also recorded as before.

### 2.5. Statistical Analysis

Results are expressed as the mean ± standard error of the mean (SEM) for each group. Data were analyzed using SPSS version 25 software ANOVA followed by the Tukey test were used. A *P* value < 0.05 was considered statistically significant.

## 3. Results

### 3.1. Preventive Effects of Single-Dose of *D. Guineense* Fruit Pulp on Castor Oil Induced Diarrhea

**Figure 1:**
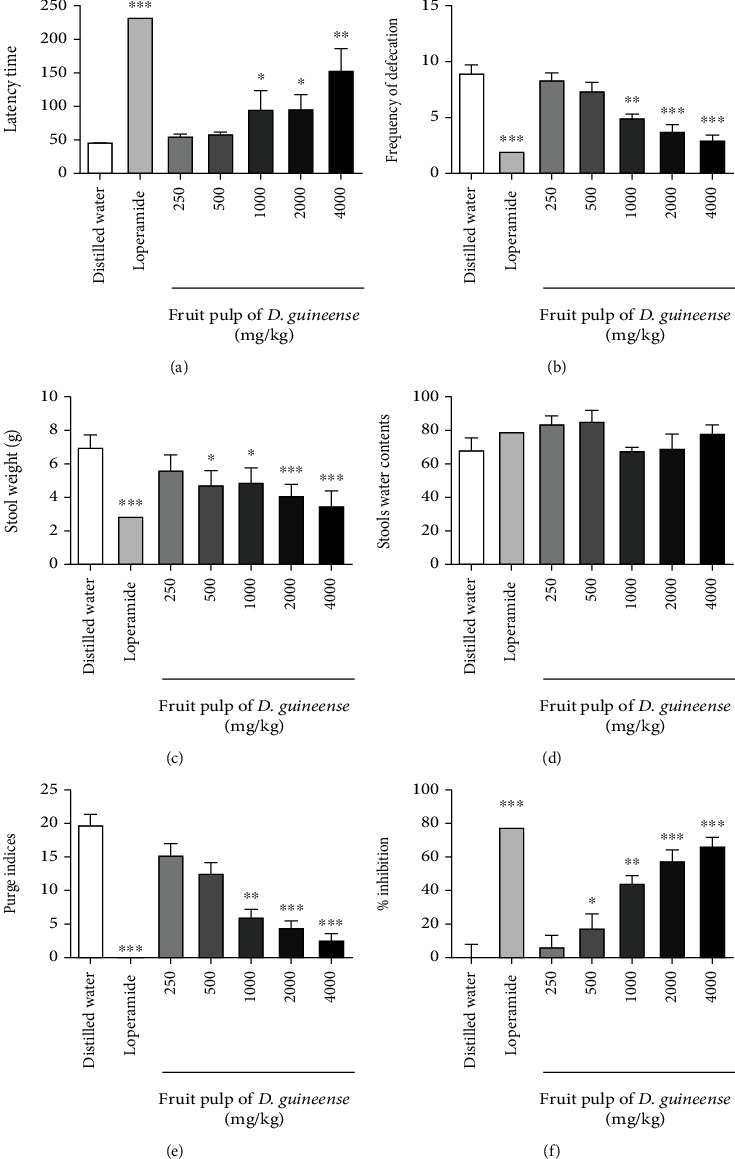
Preventive effect of single dose *D. guineense* fruit pulp on castor oil diarrhea. The results are expressed as the mean ± ESM (Standard Error on the Mean) with *n* = 5; degree of significance ^∗^*P* < 0.05, ^∗∗^*P* < 0.01, and ^∗∗∗^*P* < 0.001 compared to the negative control group (one-way ANOVA followed by Tukey's multiple comparison test).

All water and *D. guineense* pretreated rats had diarrhea, while only 40% of loperamide pretreated rats had diarrhea. *D. guineense* fruit pulp at 1000, 2000, and 4000 mg/kg, on the other hand, significantly delayed diarrhea (Figure 1(a)); reduces the frequency of defecation (Figure 1(b)) and reduces the amount of diarrheal stools (Figure 1(c)). But the water content of the stools of the batches treated with *D. guineense* does not vary significantly compared to the controls (Figure 1(d)). *D. guineense* reduces the purging index, with a degree of inhibition comparable to that of loperamide from 2000 mg/kg (Figure 1(e)). It also reduces significantly from 500 mg/kg the diarrhea induced by castor oil (Figure 1(f)).

### 3.2. Preventive Effects of Repeated Administration of *D. Guineense* Fruit Pulp on Castor Oil Induced Diarrhea

**Figure 2:**
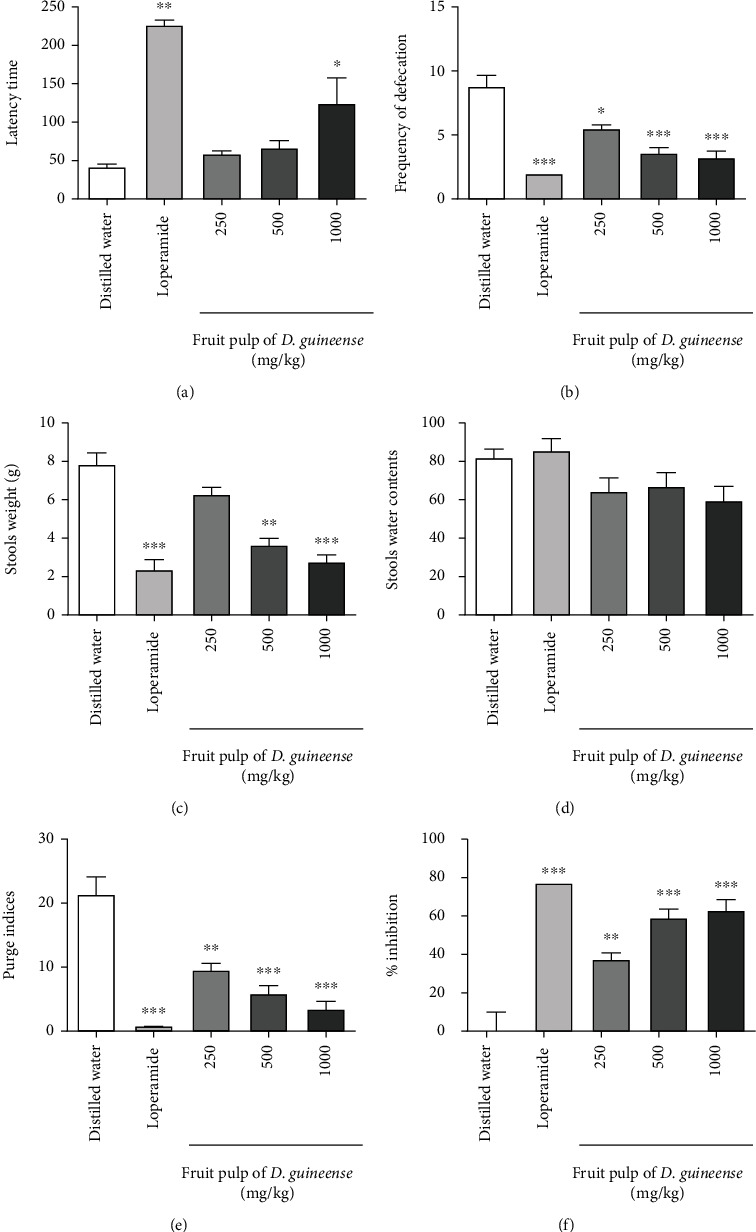
Effect of repeated administration of the fruit pulp of *D. guineense* on castor oil diarrhea. The results are expressed as the mean ± ESM (Standard Error on the Mean) with *n* = 5; degree of significance ^∗^*P* < 0.05, ^∗∗^*P* < 0.01, ^∗∗∗^*P* < 0.001 compared to the negative control group (one-way ANOVA followed by Tukey's multiple comparison test.

After 8 days of treatment, 100% of rats given 250 and 500 mg/kg of *D. guineense* fruit pulp, and distilled water had diarrhea; whereas only 80% of those who received the 1000 mg/kg pulp dose and 40% for the loperamide group responded with diarrhea. It appears that the doses of 250 and 500 mg/kg, which were not effective with the single-dose preventive test, significantly delayed diarrhea; reduces the frequency of diarrheal stools and also reduces the purging index (Figures 2(a), 2(b), 2(c), and 2(e)). The effects of *D. guineense* at a dose of 1000 mg/kg are comparable to those of loperamide (*P* < 0.001) (Figure 2(f)).

### 3.3. Curative Effects of *D. Guineense* Fruit Pulp on Castor Oil-Induced Diarrhea

**Figure 3:**
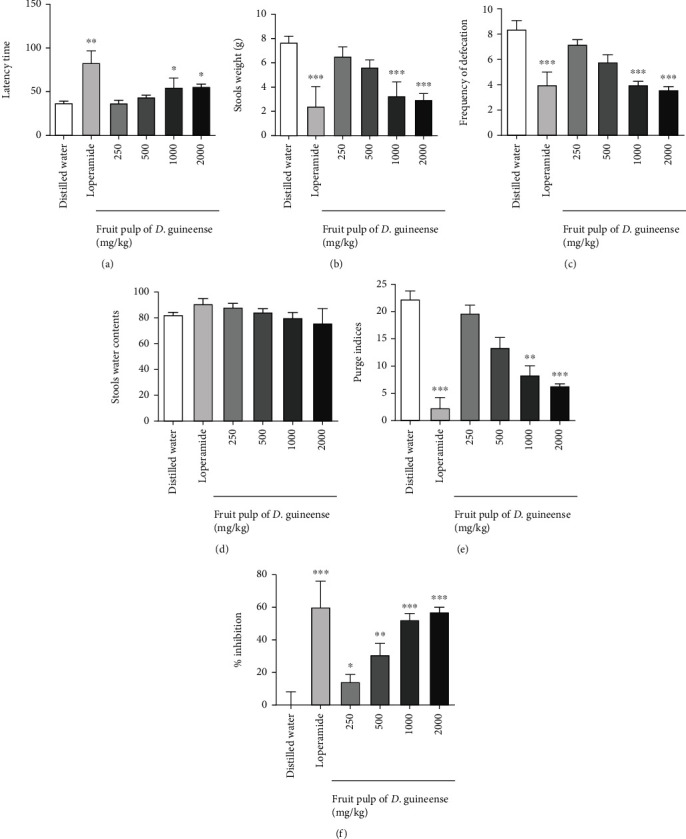
Curative effect of *D. guineense* fruit pulp on castor oil-induced diarrhea. The results are expressed as the mean ± ESM (Standard Error on the Mean) with *n* = 5; degree of significance ^∗^*P* < 0.05, ^∗∗^*P* < 0.01, ^∗∗∗^*P* < 0.001 compared to the negative control group (one-way ANOVA followed by Tukey's multiple comparison test).

A delay in the onset of diarrhea in rats having received the fruit pulp of *D. guineense* was observed with a reduction in the quantity of diarrheal stools, in the frequency of defecation, and in the purging index. The effects of *D. guineense* at a dose of 2000 mg/kg are slightly less to those of loperamide (P <0.001) (Figures 3(a)–3(d)). *D. guineense* significantly reduces from 250 mg/kg the diarrhea induced by castor oil (Figure 3(f)).

## 4. Discussion

Medicinal plants have become a worldwide topic drawing an impact on world health. Herbal medicine has played a crucial role in the maintenance of the healthcare system of the wide population throughout the world [28]. *D. guineense* is widely consumed in Togo because of the sweetness of his fruits. Our results, regarding the single-dose of effect of *D. guineense*, have showed that this pulp delayed the onset of diarrhea. On the other hand, it significantly reduced the frequency and quantity of production of wet feces. It also reduced the purge index and the degree of dehydration of the animals. These results are similar to those of Elizabeth et al. with the aqueous extracts of the leaves of this same plant [11]. The reduction in the purging index, confirms a weakening of the force of intestinal evacuation [9, 29].

Seeing the weak effects of *D. guineense* pulp during the single-dose test on diarrhea, we have conducted an 8-day test with doses of 250, 500, and 1000 mg/kg. The results revealed that this pulp, after 8 days of intake, reduced significantly the frequency of defecation, the quantity of diarrheal stools emitted as well as the purge index. *D. guineense* pulp has, therefore, a cumulative effect on the digestive tract and then can protect against diarrhea [26]. These effects are comparable to those of probiotics as reported by Vanjari et al. [26], on Wistar rats.

The results observed in the curative test are comparable to those of the single-dose protection test with a slight increase of the inhibition of diarrhea. At this test, the plant significantly delayed diarrhea from 1000 mg/kg. Therefore, the fruit pulp of *D. guineense* can be used as a medicine in the treatment of metabolic or inflammatory diarrhea. Unlike the usual tests carried out on antidiarrheal products, which consist in the administration of the product 60 min. before the induction of the diarrhea, in this study we have induced the diarrhea, by the administration of castor oil (curative test), then treated this diarrhea with our products. The results obtained confirmed the traditional use of *D. guineense* in Nigeria in the treatment of diarrhea [30]. This antidiarrheal effect observed with *D. guineense* would be due to the composition of this pulp in osmotically active molecules such as carbohydrates and minerals such as Na^+^ (main driver of gastrointestinal osmotic phenomena), Mg^2+^, and Ca^2+^ [31, 32], which would initially have created a call for water in the lumen of the gastrointestinal tract at first [32].

## 5. Conclusions

The fruit pulp of *D. guineense* has showed antidiarrheal effects in Wistar rats by reducing the frequency of defecation and the amount of diarrheal fecal matter emitted as well as the water content. It also delayed the onset of diarrhea and significantly reduced the purging index like loperamide. Although further studies are warranted by using different antidiarrheal models and solvents, the results of this study confirmed the traditional use of *D guineense* fruit pulp in the treatment of diarrhea.

## Data Availability

All data generated or analyzed during this study are included in this published article.
